# Clinical application of high‐LET radiotherapy combined with immunotherapy in malignant tumors

**DOI:** 10.1002/pro6.1225

**Published:** 2024-03-20

**Authors:** Kexin Meng, Haijun Lu

**Affiliations:** ^1^ Qingdao University Qingdao Shandong China; ^2^ Department of Radiotherapy The Affiliated Hospital of Qingdao University Qingdao Shandong China

**Keywords:** carbon ion radiotherapy, high linear energy transfer, immunotherapy, malignant tumor, photon radiotherapy, proton therapy

## Abstract

The superior physical and biological properties of high linear energy transfer (LET), as opposed to traditional low‐LET rays, underscore the advantage of proton therapy (PRT) and carbon ion radiotherapy (CIRT) are better than traditional photon radiotherapy (XRT). With the advancements in science, an increasing number of hospitals have introduced new technologies. However, radiotherapy is primarily used for local treatment, which means that if the tumor has metastasized to distant sites, it is often necessary to combine it with systemic therapies such as immunotherapy. In recent years, the combination of high‐LET radiotherapy and immunotherapy has emerged as a promising treatment option in oncology and many studies have confirmed its efficacy for both local and distant metastases. In this review, we summarize the effects of PRT and CIRT on the immune system in detail, followed by an introduction to preclinical and clinical studies of PRT and CIRT in combination with immune checkpoint inhibitor (ICIs) therapy. We also briefly introduce some preclinical studies on CIRT in combination with dendritic cells (DCs) and Treg inhibitor therapies.

## Graphical abstract

High LET radiation has a high ionization density, causing more complex aggregated DNA damage to induce greater immunogenicity potential.

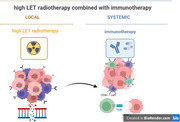

## 1 BACKGROUND

The impact of radiotherapy on the patients' immune system is like a double‐edged sword, which may not only damage local immune tissues and cause immunosuppressive effects but also induce and activate local anti‐tumor immune effects. Additional investigations have revealed the existence of the “abscopal effect.”^1^ Currently, research on radiotherapy combined with immunotherapy is underway, especially for patients with advanced distant metastasis or recurrence.^2^, ^3^ Theoretically, high linear energy transfer (LET) radiation therapy is more advantageous than photon radiotherapy (XRT) for enhancing the efficacy of immunotherapy. Contrastingly, due to the existence of Brag peaks, the dose distribution of high LET radiation is more concentrated, resulting in less damage to the immune system. However, high LET radiation has a high ionization density, causing more complex aggregated DNA damage, and is associated with greater immunogenicity potential.^4^ Several studies have shown that high‐LET radiation therapy combined with immunotherapy can synergistically promote local and systemic antitumor immune effects, resulting in favorable outcomes. Currently, the main therapies used in clinical practice are proton therapy (PRT) or carbon ion radiotherapy (CIRT) combined with immune checkpoint inhibitors (ICIs) therapies, including cytotoxic T‐lymphocyte antigen 4 (CTLA‐4), programmed cell death protein 1 (PD‐1), and programmed cell death ligand 1 (PD‐L1) inhibitor therapies.

## 2 THE EFFECTS OF PRT ON THE IMMUNE SYSTEM

PRT has a dose distribution advantage over XRT, which helps protect immune tissues and alleviate immunosuppressive effects. Photon radiation possesses substantial penetrability. While some energy is deposited along the path in the tissue, a considerable portion penetrates the entire body and subsequently leaves. While protons are charged particles, most of their energy is deposited at the Brag peak at a controllable depth. These differences indicate that the dose distribution of PRT is more concentrated in the target area. In a phase I clinical trial called “HYDRA” (NCT05364411), Elbers et al.^5^ observed that, within the context of head and neck squamous cell carcinoma (HNSCC), PRT could reduce radiation dose and volume through high‐precision radiation treatment planning and dose delivery. This approach aimed to achieve a superior immune‐sparing effect, leading to improved outcomes.

PRT results in a decrease in lymphocyte levels in patients' peripheral blood, although it is milder in comparison to XRT. This effect can be alleviated by adjusting radiation parameters, ultimately contributing to an increased sparing of lymphocytes. However, increasing the dose rate or reducing the number of fractions can benefit patients more in a patient‐specific manner. In a single‐institution, open‐label, non‐blinded, phase II randomized trial (NCT01512589) conducted between April 2012 and March 2019, Wang et al.^6^ observed that in the context of esophageal cancer, limited dose scatter by PRT significantly reduced the incidence of grade 4 lymphopenia compared with intensity modulated radiotherapy (IMRT), especially in intermediate‐risk patients who received standard adjuvant immunotherapy. Kim et al.^7^ observed reduced radiation exposure to circulating blood cells in pencil beam scanning‐proton beam therapy (PBS‐PBT) in comparison to IMRT in locally advanced lung cancer. The results of this study demonstrate that lymphocyte‐sparing radiotherapy in PBS‐PBT can minimize the risk of severe radiation‐induced lymphopenia and enhance outcomes, particularly in maintenance immunotherapy. However, McCullum et al.^8^ observed that among patients with hepatocellular carcinoma (HCC), those with highly sensitive lymphocytes benefited the most from decreasing dose rates, whereas those with slow lymphocyte recovery benefited the most from shorter fractionation regimens. Additionally, increasing the dose rate at the same fractionation reduced the absolute lymphocyte count depletion more significantly than reducing the number of fractions.

PRT induces an inflammatory response mediated by the altered expression of inflammation‐regulating cytokines, but is dissimilar to that of XRT, as the principal pro‐inflammatory cytokines undergo distinct regulation both in the short‐ and long‐term following irradiation. The early response caused by PRT is milder; therefore, normal tissue damage recovers faster, but late responses, such as fibrosis, are more severe, and the efficacy of immunotherapy can potentially be influenced. Nielsen et al.^9^ observed that the cytokines interleukin (IL)‐6, IL‐1β, IL‐10, interferon (IFN)‐γ, and tumor necrosis factor (TNF)‐α were expressed at lower levels 16 months post proton irradiation. These parameters were downregulated on day 5 and increased to the same level or higher on day 30 in comparison to the photon irradiation group, which did not show a significant change on days 5 and 30.

## 3 APPLICATION OF PRT COMBINED WITH IMMUNOTHERAPY

### 3.1 Preclinical studies

ICIs can enhance the response of tumors to PRT, although this may depend on the type of ICIs utilized. There is still ongoing controversy and debate over which type of ICIs may be more effective in this context. Nielsen et al.^10^ found that anti‐CTLA‐4 treatment enhanced the response of breast cancer mouse models to PRT, whereas neither anti‐PD‐1 nor anti‐PD‐L1 treatment significantly affected tumor control. However, Chen et al.^11^ found that the combination of PRT and anti‐PD‐L1 therapy delayed HCC tumor growth in comparison to PRT alone treatment in Hepa1‐6 syngeneic mouse models. Hu et al.^12^ observed that the combination of PRT and anti‐PD‐1 therapy reduced growth in both irradiated and non‐irradiated tumors in a two‐tumor mice model of lung cancer, and the response was further enhanced by the injection of irradiated NBTXR3 nanoparticles into tumors.

### 3.2 Clinical studies

The combination of PRT and ICIs therapy could serve as an alternative treatment for patients who cannot tolerate XRT alone. Currently, clinical research is focused on non‐small cell lung cancer (NSCLC). Carrasquilla et al.^13^ used PBS‐PBT followed by duvalizumab treatment in high‐risk patients and the results showed favorable progression‐free survival (PFS) and overall survival (OS). Simultaneously, certain studies suggest that for the majority of early‐stage patients who can receive curative radiotherapy, PRT alone is sufficient, while the combination of ICIs therapy may lead to overtreatment; therefore, it is particularly important to identify suitable patients in advance. Nakamura et al.^14^ found that pretreatment DNA analysis could be used to predict recurrence in patients with cT_1‐2_N_0_M_0_ non‐squamous NSCLC to determine whether combined ICIs therapy was necessary.

The combination of PRT and ICIs therapy is also an option for patients with advanced or distant metastatic tumors. Su et al.^15^ retrospectively reviewed 29 patients with advanced HCC who received PRT combined with anti‐PD‐1/PD‐L1 therapy between 2016 and 2019 and found it to be efficient. In addition to relevant large‐scale retrospective studies, several case reports have supported its efficacy. Examples of common metastatic sites include a study on a high‐risk 65‐year‐old male with extensive metastasis from clear cell renal cancer.^16^ Immunotherapy proved effective in treating bone metastases, whereas intensity‐modulated proton therapy (IMPT) was effective in treating pleural and thoracic lymph node metastases. Examples of rare metastatic sites include a patient with metastatic large‐cell neuroendocrine carcinoma who achieved a durable partial response following combinational treatment with proton craniospinal irradiation, bevacizumab, and pembrolizumab.^17^

The combination of PRT and ICIs therapy may also be considered a second‐line option in selected patients with recurrences, especially for isolated recurrences. Related clinical studies have primarily focused on NSCLC, whereas a few studies have focused on HNSCC. A phase II, single‐arm trial (NCT03087760) of consolidation pembrolizumab after PRT reirradiation for locoregional recurrences in 22 patients with NSCLC conducted between 2017 and 2021 showed acceptable PFS and favorable OS, with late grade 5 toxicity occurring in only 2 patients.^18^ A retrospective cohort study of patients with recurrent NSCLC treated with definitive IMPT re‐irradiation between 2019 and 2021 showed prolonged disease control with limited toxicity, particularly in combination with immunotherapy.^19^ A phase II clinical study of 31 HNSCC patients enrolled between March 2018 and July 2020 showed the combination of durvalumab/tremelimuab and PRT was tolerated well in individuals who had undergone extensive prior treatments.^20^

## 4 THE EFFECTS OF CIRT ON THE IMMUNE SYSTEM

CIRT has the potential to elicit immune activation of lymphocytes, such as T cells, NK cells, and DCs; increase lymphocyte proliferation; enhance lymphocyte functionality; limiting the induction of immunosuppressive cells such as myeloid‐derived suppressor cells (MDSCs); and reduce the expression of immunosuppressive cytokines. Hu et al.^21^ assessed the immune response evoked by CIRT in 32 patients with localized prostate cancer, and the results showed that there were no significant differences in the frequencies of CD3 ^+^, CD4 ^+^, CD8 ^+^T cells, and NK cells, whereas the CD4/CD8 ratio increased in all lymphocyte subsets except regulatory T cell (Tregs) proliferation and T cell functionality enhancement. However, we did not observe any changes in the MDSCs. Animal experiments have also shown increased infiltration of CD8^+^T cells into the tumor microenvironment after CIRT,^22^, ^23^ one of which even showed a decreased influx of MDSCs^23^. Wang et al.^24^ found that CIRT induces Klrk1 gene expression and activates the NKG2D/NKG2D‐Ls pathway, thereby improving the infiltration and functional status of NK cells. A study by König et al.^25^ on DCs found that phenotypic maturation remained unchanged after carbon ion irradiation, and functionalities such as phagocytosis, migration, and cytokine secretion remained unaffected, indicating a possible persistent potential for inducing an adaptive immune response.

CIRT has greater immunogenic potential than XRT. Zhou et al.^22^ found that CIRT triggered immunogenic cell death more efficiently, including exposure to calreticulin, the release of adenosine triphosphate (ATP), and the release of high‐mobility group box 1 (HMGB1). Huang et al.^26^ observed that all three types of radiation, including photons, protons, and carbon ions, could increase calreticulin exposure in a time‐dependent manner; however, carbon ion radiation required the smallest dose and achieved the best efficacy. Studies have also observed a greater release of tumor HMGB1 after CIRT than after XRT.^27^, ^28^, ^29^

## 5 APPLICATION OF CIRT COMBINED WITH IMMUNOTHERAPY

### 5.1 Preclinical studies

Preclinical studies have demonstrated the efficacy and superiority of CIRT in combination with ICIs therapy. First, it efficiently inhibits both local and distant tumors. Takahashi et al.^27^ found that CIRT combined with dual immune checkpoint blockade therapy (anti‐PD‐L1 and anti‐CTLA‐4 antibodies) delayed the growth of both irradiated and non‐irradiated tumors in osteosarcoma mouse models. Second, CIRT was superior to XRT combined with ICIs therapy. Helm et al.^30^ found that the combination of CIRT and ICIs resulted in a more significant delay in distant tumor growth in osteosarcoma mouse models than sequential XRT and ICIs therapy. Third, the study by Huang et al.^31^ demonstrated the superiority of the “1+1>2” effect, which is superior to CIRT or ICIs therapy alone. Zhou et al.^22^ demonstrated two advantages simultaneously: CIRT improved the efficacy of anti‐PD‐1 treatment in melanoma mouse models, and the improvement was superior to that of XRT.

Preliminary explorations were conducted to elucidate the mechanism of combination therapy. Hartmann et al.^32^ observed the combination of carbon ions and CTLA‐4 inhibitors reshaped the tumor‐infiltrating immune cell composition, activated NK cells and tumor‐associated macrophage clusters, upregulated the expression of TNF‐α and IL‐1 responsive genes, and induced systemic immunological effects in non‐irradiated tumors. Thus, better outcomes can be achieved. Huang et al.^33^ observed that carbon ions induced higher expression of calreticulin and PD‐L1 under anoxic conditions in comparison to photon or proton radiation. Therefore, it can be predicted that CIRT combined with anti‐PD‐L1 therapy may achieve better outcomes.

Additionally, preclinical studies have been conducted on the combination of CIRT with DCs or Treg inhibitor therapies. The combination of CIRT and DCs therapies can inhibit tumor metastasis. Combination therapy with CIRT and intravenous injection of DCs showed advantages in mouse models of metastatic lung cancer and was superior to the combination of XRT or intratumoral injection of DCs.^34^ However, the genetic background of the host may have a strong effect on its potency, as a study found that CIRT combined with immature DCs therapy could effectively repress distant lung metastasis in C57BL/6J and C3H/He mouse models but did not show enhancement of metastasis suppression in BALB/c mouse models.^35^ Preclinical studies on the combination of CIRT and Treg inhibitor therapy are ongoing. Wang et al.^24^ found that CIRT combined with Treg inhibitor therapy enhanced the infiltration and function of NK cells in the tumor microenvironment of lung cancer and prolonged the survival time of mice.

### 5.2 Clinical studies

Clinical studies have evaluated or are currently evaluating the safety, efficacy, and superiority of CIRT combined with ICIs therapy. A multicenter, open‐label, nonrandomized phase II clinical trial called “ICONIC” (NCT05229614) aims to assess the feasibility and clinical activity of the combination of CIRT and ICIs therapy in 27 patients who have attained disease stability with pembrolizumab treatment.^36^ The primary endpoint is the objective response rate, and the secondary endpoints are safety, survival, and disease control rates. If the results are statistically significant, this will be the first evidence of related research. Another study analyzed the adverse events (AEs) of sequential CIRT and ICIs in patients with advanced melanoma. The results showed that the frequencies of early and late AEs were 100% and 82%, respectively, and the frequency of G3+ AEs was in line with previous literature.^37^

## 6 SUMMARY AND PROSPECT

Theoretically, owing to the unique biological and physical properties of high‐LET rays, immunotherapy combined with PRT or CIRT can achieve better results than XRT, improving control over local and distant tumors and reducing the occurrence of side effects. Several preclinical and clinical studies have demonstrated this finding. However, this approach is still far from mature in clinical practice. However, the evidence provided by preclinical studies is not convincing. As for clinical research, they mainly focus on limited types of NSCLC, limiting the applicability to other malignant tumors. Second, the patients studied are not representative, with most of them either not being able to tolerate the treatment or demonstrating poor outcomes after XRT. Additionally, there is a lack of early and initial treatment patients. Third, due to the lack of particle radiotherapy centers and unified standards, relative research is not sufficient to reach a consensus. Therefore, further research is needed to ascertain whether the combination of high LET radiation and immunotherapy can genuinely yield significant improvements in practical applications. It is essential to broaden the indications and formulate specific treatment plans to truly enhance the control or even cure rate of malignant tumors.

## CONFLICT OF INTEREST STATEMENT

The authors declare that they have no conflict of interests to disclose.

## ETHICS STATEMENT

Not applicable.
